# Paget's disease of the breast in a male with lymphomatoid papulosis: a case report

**DOI:** 10.1186/1752-1947-5-43

**Published:** 2011-01-28

**Authors:** Dina Fouad

**Affiliations:** 1 Aberdeen Royal Infirmary, University of Aberdeen, Scotland

## Abstract

**Introduction:**

Paget's disease is an eczematous skin change of the nipple that is usually associated with an underlying breast malignancy. Male breast cancer represents only 1-3% of all breast malignancies and Paget's disease remains very rare.

**Case presentation:**

We present the case of a 67-year-old Caucasian man with lymphomatoid papulosis who was diagnosed with Paget's disease of the nipple and who was treated successfully with surgery alone. We discuss the presentation, investigations, management and pathogenesis of Paget's disease of the nipple.

**Conclusion:**

The case highlights the need to be vigilant when new skin lesions arise in the context of an underlying chronic skin disorder.

## Introduction

Paget's disease is an eczematous skin change of the nipple that is usually associated with an underlying breast malignancy [1]. It may present with erythema, scaling, ulceration, bleeding or a painful nipple [2,3]. Male breast cancer accounts for less than 1% of all breast cancer with Paget's disease remaining very rare. Paget's disease of the nipple may be associated with an underlying invasive cancer, a non-invasive cancer ductal carcinoma *in situ *or no underlying cancer. Prognosis is dependent upon the status of invasion and treatment is tailored accordingly. Approximately 90% of patients presenting with a palpable mass or who have a visible mass on mammography will have underlying invasive disease. Notably, invasive cancer can occur with Paget's disease in 38% of patients with no underlying mass [3,4].

## Case Presentation

The patient is a 67-year-old Caucasian man who presented to the Breast Clinic in August 2008 with a six-month history of a painful right nipple and one episode of clear nipple discharge. His problem had not resolved with use of a topical ointment prescribed by his general practitioner and he was admitted to the Breast ward of our hospital in September 2008 for further investigations.

The patient's past medical history includes 30 years of lymphomatoid papulosis, a chronic papulonodular dermatological condition, which has been controlled with long-term methotrexate treatment and folic acid supplementation. There was no report that the control of this had been particularly poor recently, however the patient had several previous recorded flare ups (1992, 2000, 2004, 2006) requiring clinic appointments and adjustment of medication (mainly methotrexate).The patient has also suffered from essential hypertension, atrial fibrillation and atrial flutter since 1990 for which he takes bendrofluamethiazide and digoxin respectively. In addition, the patient was diagnosed with mixed cellularity Hodgkin's lymphoma nine years ago (1999) and successfully treated with six cycles of combination chemotherapy (ABVD: doxorubicin, bleomycin, vincristine and dacarbazine), being in remission to date. The lymphoma was discovered on palpation of two left sided inguinal nodes, one right sided inguinal node, palpable lumps in the left upper thigh, left lower quadrant of the abdomen and the right hypochondrium. A computed tomography (CT) scan revealed retroperitoneal lymphadenopathy, bilateral inguinal lymphadenoapathy and nodes present in the both iliac chains. The patient received no radiotherapy for this disease or for any other reason. Moreover, the patient has been extensively investigated for ongoing neurological symptoms that include paraesthesia of hands and left foot and some gait imbalance but the aetiology remains unexplained to date. The only positive family history is of a sister who died aged 68 from an unknown cancer.

On examination, the right nipple appeared inflamed, mildly erythematous and thickened with tenderness on palpation. The erythema, inflammation and thickening did not extend further than the nipple-areolar region. There was no obvious nipple inversion, masses, ulceration or active nipple discharge and no axillary or supraclavicular lymphadenopathy were palpable. Notably, faded scattered, pale pink, papules were visible across the upper chest, upper back and lower abdomen.

The patient had a mammogram, which was normal, and he proceeded to have a punch biopsy. The result of this confirmed Paget's disease of nipple and the patient was scheduled for a right mastectomy and sentinel node biopsy.

**Figure 1:**
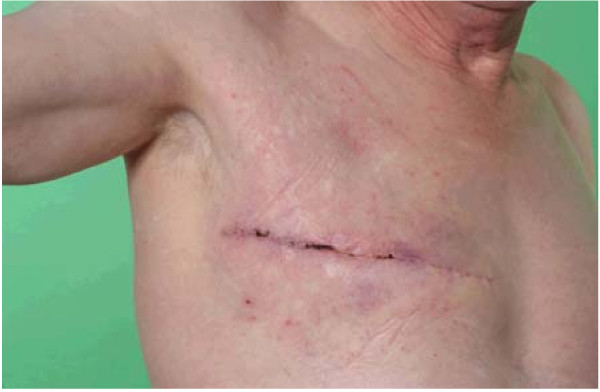
**Patient two weeks post right-sided mastectomy for Paget's disease**. Medical illustration, University of Aberdeen.

The mastectomy was uneventful and he recovered well post-operatively (Figure 1). Histopathology confirmed Paget's disease of the right nipple with no evidence of underlying invasive ductal carcinoma, ductal carcinoma *in situ *of the breast tissue or lymph node invasion.

## Discussion

Paget's disease is an eczematous skin change of the nipple that is usually associated with an underlying breast malignancy [1]. It may present with erythema, scaling, ulceration, bleeding or a painful nipple [2,3]. The condition was first described in 1874 by the surgeon, Sir James Paget, who noted that the chronic eczematous rash of the nipple preceded an underlying intraductal carcinoma [1].

Male breast cancer accounts for less than 1% of all breast cancer and Paget's disease represents 1-3% of all breast malignancies, having a higher incidence in males (5%) than females (1-4%) [3,4]. Paget's disease may present concomitantly with an underlying invasive carcinoma, ductal carcinoma *in situ *or with no underlying breast cancer. Forty six percent of Paget's cases present without a mass and of these, underlying invasive breast cancer is usually found in only 38% with ductal carcinoma *in situ *being found in the majority [4,5].

The patient had no obvious risk factors for breast cancer such as testicular abnormalities, infertility, obesity, cirrhosis or Klinefelter's syndrome nor was he known to be positive for any BRCA2 mutations [4]. However, the patient may have been at increased risk of malignancy due to long term methotrexate treatment. methotrexate has anti-folate effects and studies have shown there to be an increased risk of malignancy in those deficient of folic acid [6].

Clinical examination of the breast is usually followed by imaging, either mammography or ultrasound. Imaging may show subareolar microcalcifications, architectural distortion or nipple changes such as thickening [7]. Imaging is followed by fine needle aspiration cytology or punch biopsy. Histology may reveal hyperkeratosis, parakeratosis or acanthosis of the epidermis and infiltration with the classical Paget cell that is large, ovoid, has pale staining cytoplasm and hyperchromic nuclei [1,2].

The pathogenesis of Paget's disease is still a subject of debate with two main hypotheses. The epidermotropic hypothesis proposes that Paget's cells originate from ductal epithelium, from where they migrate towards the epidermis. This hypothesis is supported by the association between Paget's and an underlying breast carcinoma in the majority of patients. The second hypothesis, the intraepidermal transformation theory, considers the presence of malignant keratinocytes that originate from the areolar epidermis. Our case supports this origin since there was no underlying carcinoma [8,9].

Treatment is usually a mastectomy plus axillary node sampling or clearance. Adjuvant treatment may be considered depending on nodal and receptor status [3]. Breast conservation surgery with radiotherapy, or radiotherapy alone, are not usually considered due to high recurrence rates [8]. However, studies have shown breast conserving surgery to be a feasible and safe option [10-12]. The prognosis of Paget's depends on the presence of an invasive cancer and axillary lymph node spread. This case is stage 0 as there is no underlying breast malignancy or lymph node spread and the five-year survival is 92-94% [9].

Several differential diagnoses should be considered when Paget's disease is suspected including malignant melanoma, pagetoid dyskeratosis, Bowen's disease and inflammatory skin conditions of the nipple e.g. seborrhoeic dermatitis, contact dermatitis, post-radiation dermatitis, eczema and psoriasis [5]. This patient has lymphomatoid papulosis, a condition in which groups of pruritic papules at different stages of development recurrently arise mainly on the trunk and limbs. It is conceivable that the papulosis may have masked his Paget's nipple lesion and delayed its diagnosis. Moreover, research has shown that lymphomatoid papulosis and Hodgkin's disease along with cutaneous T-cell lymphoma are all connected, being derived from the same T-cell clone [13]. Cases have been reported of patients developing lymphomatoid papulosis, followed by Hodgkin's disease and lastly developing cutaneous T-cell lymphoma [9]. Therefore this man may be at high risk for cutaneous T-cell lymphoma, which can present as erythematous patches resembling eczema. It is essential that the patient is monitored closely.

## Conclusion

The case highlights the need to be vigilant when new skin lesions present in the context of an underlying chronic skin disorder.

## Consent

Written informed consent was obtained from the patient for publication of this case report and accompanying images. A copy of the written consent is available for review by the journal's Editor-in-Chief.

## Competing interests

The authors declare that they have no competing interests.

## Authors' contributions

DF performed the literature search, gathered and analysed the relevant test results and wrote the report. EEO reviewed the manuscript. The author approved the final manuscript prior to submission.
